# Can We Prevent Antimicrobial Resistance by Using Antimicrobials Better?

**DOI:** 10.3390/pathogens2020422

**Published:** 2013-06-10

**Authors:** Germander Soothill, Yanmin Hu, Anthony Coates

**Affiliations:** 1 Barts and The London School of Medicine and Dentistry, Queen Mary, University of London, Garrod Building, Turner Street, Whitechapel, London, E1 2AD, UK; E-Mail: g.soothill@smd10.qmul.ac.uk; 2 Medical Microbiology, Centre for Infection, Division of Clinical Studies, St George’s, University of London, Cranmer Terrace, London SW17 0RE, UK; E-Mail: ymhu@sgul.ac.uk

**Keywords:** antimicrobial resistance, pharmacokinetics, pharmacodynamics, minimum inhibitory concentration (MIC), mutant prevention concentration (MPC), microbiota, colonisation resistance, antibiotic combination, bacteria-antimicrobial pairing, antimicrobial cycling

## Abstract

Since their development over 60 years ago, antimicrobials have become an integral part of healthcare practice worldwide. Recently, this has been put in jeopardy by the emergence of widespread antimicrobial resistance, which is one of the major problems facing modern medicine. In the past, the development of new antimicrobials kept us one step ahead of the problem of resistance, but only three new classes of antimicrobials have reached the market in the last thirty years. A time is therefore approaching when we may not have effective treatment against bacterial infections, particularly for those that are caused by Gram-negative organisms. An important strategy to reduce the development of antimicrobial resistance is to use antimicrobials more appropriately, in ways that will prevent resistance. This involves a consideration of the pharmacokinetic and pharmacodynamics properties of antimicrobials, the possible use of combinations, and more appropriate choice of antimicrobials, which may include rapid diagnostic testing and antimicrobial cycling. Examples given in this review include *Mycobacterium tuberculosis*, Gram-negative and Gram-positive organisms. We shall summarise the current evidence for these strategies and outline areas for future development.

## 1. Introduction: The Problem of Antimicrobial Resistance

The theme of World Health Day 2011 *“antimicrobial resistance: no action today*, *no cure tomorrow”* highlighted antimicrobial resistance as a major issue. Boucher *et al.* describe the pathogens currently presenting the biggest problem in terms of antimicrobial resistance as the ESKAPE pathogens: *Enterococcus faecium*, *Staphylococcus aureus*, *Klebsiella pneumoniae*, *Acinetobacter baumanii*, *Pseudomonas aeruginosa*, and *Enterobacter species* [1,2]. The detection of the Metallo-beta-lactamase (NDM-1) enzyme in 2008 alarmed the international community [3,4]. There is a lack of treatment options to deal with some of the multidrug resistant pathogens. The result is the use of (i) last-line therapies such as carbapenems, that would ideally be preserved [5]; and (ii) antimicrobials previously discarded due to toxicity, such as polymyxins, which have been brought back into use despite nephrotoxicity and neurotoxicity [6].

The development of novel antimicrobials is a subject of considerable interest, which has been discussed in depth by commentators [7,8,9,10,11,12]. However, new drug development alone cannot solve the problem. With few new classes of antimicrobials currently in the pipeline, and others that are still years away from the market, resistance prevention strategies have a crucial role to play in preserving the lifespan of existing antimicrobials [13,14]. 

Strategies to extend the lifespan of the antimicrobials by preventing antimicrobial resistance include: i) reducing usage of antimicrobials; ii) appropriate use of antimicrobials; and iii) infection control [15]. According to the World Health Organisation, limiting the development of antimicrobial resistance ultimately lies with reducing antimicrobial usage [16]. Reducing antimicrobial usage requires tackling a number of difficult areas, including reducing over-the counter purchases, public health campaigns, education of medical professionals, and reducing antimicrobial usage in animals, as has been discussed by excellent reviews on the subject [5,17,18,19,20,21]. Preventing cross-infection with resistant bacteria prevents the spread of antimicrobial resistant infections and so infection control is an important strategy, including outbreak control, hand hygiene, barrier precautions, and prophylactic antimicrobials, as discussed by reviews [13,16,22,23]. This review will focus on the second core strategy, to extend the lifespan of current antimicrobials by using the ones we have in ways that will reduce the emergence of resistance. 

## 2. Appropriate Antimicrobial Usage

Clinically relevant resistance due to chromosomal mutations develops gradually due to the accumulation of alterations [24]. The mutation rate differs between antimicrobial-bacteria pairings because some antimicrobials select resistant mutations in certain bacteria more readily. Choice of antimicrobial-bacterial pairing, antimicrobial combinations, and appropriate dosing can potentially reduce the emergence of *de novo* resistance. A good example of this is treatment of *Mycobacterium tuberculosis*, in which resistance arises solely by chromosomal mutations (the successful use of combination therapy to avoid resistant mutants for *Mycobacterium tuberculosis* is discussed in section 2.2.). In contrast, the transfer of resistance genes, for instance via plasmids, can confer clinically relevant resistance in a single step. Slowing the progress of this type of resistance is extremely difficult [24]. This type of resistance transfer is particularly common in multi-species populations in the large intestine in humans. Horizontal gene-transfer accounts for much of resistance to antimicrobials in hospital-acquired infections for the majority of antimicrobial classes [25].

### 2.1. Pharmacokinetics and Pharmacodynamics

Pharmacokinetics and pharmacodynamics are topics of great importance in the areas of anti-bacterial efficacy and clinical outcomes [26]; recently they have also been applied to the topic of antimicrobial resistance.

#### 2.1.1. Dosage

Progress in the understanding of how the dose of antimicrobials affects resistance has been reviewed by Olofsson *et al* [27]. They describe how single high doses might be better at preventing resistance than multiple low doses (as shown in both *in vitro* and *in vivo* models [28,29,30,31,32,33,34]). Further research is required to highlight the optimal doses, which will successfully treat bacterial infections while preventing resistance. 

Consensus has not yet been reached regarding which parameter of dose assessment should be used in resistance studies. Minimum inhibitory concentration (MIC) is the minimum concentration of an antimicrobial that will inhibit the growth of bacteria after an appropriate period of incubation [26]. Blaser *et al.* have showed that maximising the ratio of maximum concentration in serum (C_max_) to MIC restricted the growth of resistant mutants in *in vitro* studies [28]. Moreover, Thomas *et al.* have suggested that selection of antimicrobial resistance is decreased if the ratio of the area under the curve (AUC) to MIC is greater than 100 [35]. Traditionally, the efficacy of anti-tuberculosis antimicrobials has been associated with a high AUC to MIC ratio. However, in contrast, it has recently been shown that in the case of *Mycobacterium tuberculosis* treatment, the efficacy of isoniazid and rifamycins may be determined by peak drug concentrations [36]. More research in this area is needed but, if this concept is confirmed, then high doses of rifamycins may be able to shorten tuberculosis treatment. It is anticipated that shortening the duration of tuberculosis chemotherapy should lead to an increase in patient adherence [37,38]. 

Blondeau *et al.* examined the effect of using high dose fluroquinolones against *Streptococcus pneumoniae in vitro* studies [39]. They used concentrations of fluroquinolones equal to the mutation prevention concentration (MPC), concentration above which no first step resistant mutants grow, in order to slow the development of fluroquinolone resistance. *In vivo* studies in rabbits show that the use of a levofloxacin concentration greater than MPC supresses the growth of mutant populations in *Staphylococcus aureus*, and use of a vancomycin concentration above MPC or with a high AUC to MIC ratio reduces the acquisition of resistance in *methicillin-resistant Staphylococcus aureus* [40,41]. Whether dosing to achieve the MPC will prevent resistance development in human patients needs more investigation. Since each antimicrobial-bacterial pairing has a unique MPC, it may not always be possible to achieve the MPC with a safe antimicrobial dose [42]. Recent clinical trial data with linezolid show efficacy in extensively drug-resistant tuberculosis, however 82% of patients experienced clinically significant adverse events [43]. Although it is bacteriostatic *in vitro*, it reaches high concentrations in the lungs, which somewhat blurs bacteriostatic/bactericidal relevance *in vivo*. This also alludes to the potential role of MPC in reducing resistance. More research is needed to ascertain which parameter and levels of dose are optimal to prevent antimicrobial resistance [44,45]. Mathematical models are being used to examine the theoretical basis for shorter treatment courses and optimise the duration and dosing of antimicrobials, which will minimise the duration of symptomatic infection as well as the selection pressure for resistance. Recent conclusions from these studies are that long durations of antimicrobial therapy are less appropriate for self-limiting infections and that the potential for selection for resistance in commensal flora should be taken into account when choosing the duration of therapy [46].

#### 2.1.2. Distribution and Excretion

The site of infection and infecting bacteria are not the only considerations for resistance development. Normal human microbiota act as a barrier against colonisation by potentially pathogenic microorganisms or overgrowth of present organisms: this is known as “colonisation resistance”. Antimicrobials can detrimentally affect this microbiota, first by causing resistance, which can then be transferred by resistance genes to other microorganisms, and secondly by damaging colonisation resistance and allowing colonisation by pathogenic microorganisms or overgrowth of present microorganisms, which may be resistant. The degree of disturbance an antimicrobial may cause to microbiota depends on its spectrum, route of administration, degree of absorption, route of elimination, and inactivation either enzymatically or by binding. Using antimicrobials that cause minimal disturbance to the normal human microbiota reduces the risk of emergence and spread of resistance strains between patients and the transfer of resistance genes between microorganisms [47]. 

Antimicrobials are distributed throughout the body and are present in different tissues (respiratory tract, urinary tract, digestive tract) at differing concentrations. It is difficult to measure the concentrations of drugs at different sites simultaneously so *in vitro* models have tried to estimate this [48,49,50]. Animal models are also used to look at tissue distribution in major organs (lungs, spleen, kidneys, intestines, *etc.*) and data often correlates to explain efficacy at site of infection or guide the selection of formulations to improve drug disposition. The microbiota present in these sites are an important source of resistance development, especially as the bacteria in these locations are usually present in large numbers and the antimicrobials may be low in concentration. Low concentrations of antimicrobials are often associated with the development of resistance [33,51,52].

Oh *et al.* investigated the effect of the administration of clindafloxacin (a broad-spectrum quinolone) on intestinal microbiota in 12 human volunteers, who could be considered to be antibiotic naïve [53]. Oral administration resulted in high antimicrobial levels in the faeces of patients, and there was significant emergence of clindafloxacin resistant Gram-negative bacilli. As a result of their findings, Oh *et al.* suggested the restricted use of clindafloxacin. A comparison of resistance development to antimicrobials that are mainly excreted in bile, such as clindafloxacin, with those which are mainly renally excreted, such as amoxicillin, has been made and it has been suggested that there is more resistance development against those which are excreted in bile due to the affect on the microbiota in the large bowel [54]. This is an important area for future research that could inform future drug development and use of current antimicrobials. Rashid *et al.* compiled data on the ecological impact of new antimicrobial agents (ceftobiprole, ceftarolone, telavancin, dalbavancin, and tigecyclin) on the gastrointestinal microbiota of healthy volunteers and found them to have only minor ecological effects [47]. Ertapenem is primarily renally excreted and was expected to have a small affect on gut microbiota. However, a study of 10 healthy volunteers where ceftriaxone and ertapenem were administered intravenously once daily at dosages 1 g and 2 g respectively found considerable ertapenem in faeces and its impact on gut microbiota was similar to that of ceftriaxone [55]. A clinical trial of adults with complicated intra-abdominal infections, treated with piperacillin-tazobactam (3.375 g every 6 hours), or ertapenem (1 g once a day) for 4 to 14 days, were compared for bowel colonisation with resistant bacteria. Lower acquisition of resistant Enterobacteriaceae was found with ertrapenem [56].

Orally administered sustained-release formulations can be used to reduce the frequency of dosing required for antimicrobials with relatively short half-lives [57]. Orally administered antimicrobials are predominantly excreted in bile and sustained-release formulations result in an even higher level of drug in the colon and a greater potential for resistance to develop. The effect of orally administered sustained-release delivery systems on the development of resistance in the microbiota of rats has been studied and it was found that oral sustained-release amoxicillin resulted in more resistance [58].

Goldstein reviewed the effects of antimicrobials on gut microbiota in the hospital environment [59]. As opposed to the healthy volunteers in the studies conducted by Oh *et al.*, the patients discussed here could be considered to be antibiotic non-naïve. *Clostridium difficle* infection was highlighted as a growing problem in hospitals worldwide, particularly due to the disruption of gut microbiota that occurs with the use of broad-spectrum antimicrobials. Infection control measures and antimicrobial stewardship were outlined as two important strategies for *Clostridium difficle* infection prevention. Another important example discussed was the risk of intestinal colonization with resistant bacteria in patients receiving antimicrobials for intra-abdominal infections. Optimizing Intra-Abdominal Surgery with Invanz studies (OASIS-I and OASIS-II) suggest that resistant Enterobacteriaceae is less like to develop in patients treated with ertapenem compared to piperacillin/tazobactam. Furthermore, recent studies suggest the increased use of non-pseudomonal carbapenems (for example, ertapenem) does not decrease the susceptibility of *Pseudomonas aeruginosa* to the pseudomonal carbapenems (such as imipenem and meropenem). Indeed, increased ertapenem and decreased imipenem use in one study showed increased susceptibility of *Pseudomonas aeruginosa* to imipenem. 

Examples of resistance gene transfer during therapy involving microbiota organisms have been identified. They include the isolation of *Klebsiella pneumoniae* carbapenemase (KPC) 3–producing *Escherichia coli* from a carrier of KPC-3–producing *Klebsiella pneumonia*, where the patient's gut flora contained a carbapenem-susceptible *Escherichia coli* strain isogenic with the KPC-3–producing isolate, suggesting horizontal interspecies plasmid transfer [60]. Also the isolation of *Staphylococcus epidermis* and *methicillin-resistant Staphylococcus aureus*, both expressing a plasmid expressing resistance gene mupA, suggests a transfer of resistance gene mupA from *Staphylococcus epidermis* to *methicillin-resistant Staphylococcus aureus* during a trial of mupirocin for nasal decolonization [61].

A greater understanding of microbiota in the body is required to understand the true impact of antimicrobials on microbiota and the generation of resistance. Much is known about the oral microbiota, with around 700 species identified [62]. However, only 50% of the organisms are culturable so techniques such as pyro-sequencing analysis are being used to study the oral microbiota in more detail [63]. Culture-independent techniques have also enhanced the understanding of microbiotal communities in the colon [64]. Knowledge about skin microbiota is currently mostly from culture based studies with only a few culture-independent studies reported [65,66]. Culture-independent approaches have an important role to play in the understanding of human microbiota, which is necessary to increase our understanding of the development of resistance there.

### 2.2. Combination Therapy

The use of combination therapy is common in clinical practice for a number of reasons. First, preventing antimicrobial resistance. The regimen for which the use of combination therapy to prevent bacterial resistance is best established is the treatment of *Mycobacterium tuberculosis*. The British Medical Research Council, led by Denis Mitchison and Wallace Fox, pioneered multi-drug regimens for tuberculosis using the first randomised clinical trials [67,68]. This concept was extended to treatment of leprosy, human immunodeficiency virus, and cancer. Combinations are used in these cases because resistance to agents develops relatively easily. Such combinations have been most successful, since *Mycobacterium tuberculosis* develops resistance by chromosomal mutations, rather than by plasmid transfer, and since the organism lives on its own in the tissues, and does not rely of the transfer of resistance from other bacterial species. Other examples of combinations that are used to delay resistance development to the individual components are co-trimoxazole (sulphonamide and trimethoprim); lincomycine and spectinomycine; and aminoglycosides and colistin [69].

Second, some antimicrobials enhance one another’s anti-bacterial activity: in other words are synergistic. An example of such a combination is the use of penicillin and gentamicin for bacterial endocarditis. 

Third, when a critically ill patient is admitted with suspected septicaemia, and the pathogen is unknown at that stage, doctors treat with several antimicrobials in order to broaden the spectrum of species of bacteria that are targeted [70]. For instance, a combination of a beta-lactam and an aminoglycoside may be used.

Fourth, combinations can specifically target resistant bacteria. For example, the addition of clavulanic acid, which is a beta-lactamase inhibitor, to amoxicillin, renders the amoxicillin active against some beta-lactamase producing bacteria. For instance, Dagan *et al.* compared amoxicillin with amoxicillin plus clavulanic acid (Augmentin) for the treatment of non-bullous impetigo [71]. *Staphylococcus aureus* was isolated from all patients and it was found that all isolates of *Staphylococcus* were sensitive to Augmentin but resistant to amoxicillin. The clinical response was significantly better in the Augmentin recipients. Bacteria produce many different kinds of beta-lactamases and so, potentially, this is an on-going approach for the enhancement of old antibiotics. For example, recent *in vivo* studies in mice show efficacy of meropenem/clavulanate and amoxicillin/clavulanate combinations against replicating and non-replicating *Mycobacterium tuberculosis* [72].

Fifth, if dormant bacteria are important in an infection. For example, the addition of anti-dormancy agents such as pyrazinamide and rifampicin to combinations for the treatment of tuberculosis has shortened the duration of chemotherapy from 18 to 6 months [38].

A meta-analysis (including data from eight randomised controlled trials) that compared aminoglycoside/beta-lactam combination therapy with beta-lactam mono-therapy to observe the emergence of antimicrobial resistance found that aminoglycoside/beta-lactam combination therapy was not associated with reduced development of resistance when compared with beta-lactam therapy alone [73].

Animal model studies in the 1980s suggested that the addition of an aminoglycoside to beta-lactam therapy might prevent the development of resistance, particularly in the treatment of *Pseudomonas aeruginosa* [74,75]. Human trials in patients with cystic fibrosis also showed that *Pseudomonas aeruginosa* resistance occurred less frequently during combination compared to single therapy [76]. The high frequency of chromosomal mutations in *Pseudomonas* spp. was suggested to support the role of combination therapy [75]. As well as prevention of resistance, suggested benefits of combination therapy for *Pseudomonas aeruginosa* include antimicrobial synergy and improved adequacy of empirical therapy. The benefits of combination therapy must be balanced against the risk of creating multi-drug resistance organisms [77]. This may be of particular concern if the effect of agents on bacteria, initially not involved in the pathogenesis, results in horizontal gene transfer of resistance genes to the pathogens.

Combination therapy for the treatment of *Neisseria gonorrhoeae* has recently been suggested as potentially reducing the development of antimicrobial resistance but we could find no controlled study data on this [78]. Bonhoeffer *et al.* used a mathematical model to assess whether combination therapy might be useful in the treatment of *Neisseria gonorrhoeae* and concluded that resistance might be preventable with more widespread use of combination therapy [79].

### 2.3. Choice of Antimicrobial

#### 2.3.1. Bacteria-Antimicrobial Pairings

The rates of spontaneous resistance mutations for bacteria-antimicrobial pairings at certain antimicrobial concentrations are known. By considering the bacterial loads in different infected lesions, it is possible to estimate whether or not resistance to an antimicrobial is likely to develop. In some clinical situations, the probability of resistant variants being selected is so high that certain antimicrobials may be best avoided [42]. Bacteria-antimicrobial pairings that are considered high risk for the development of resistance due to *de novo* mutations include rifampicin and all species of bacteria; quinolones and *Staphylococcus aureus* or *Pseudomonas aeruginosa;* and cephalosporins and *Enterobacter* spp. or *Citrobacter* spp [26]. In contrast to rifampicin, for example, it has been suggested that antimicrobials that target the cell membrane may induce less resistance [80]. Multi-targeting *versus* single-target antimicrobials is another exciting area of investigation [81].

#### 2.3.2. Rapid Diagnostic Techniques

Narrow-spectrum antimicrobials should be used instead of broad-spectrum antimicrobials where possible to avoid affecting microbiota. Use of more narrow-spectrum antimicrobials may reduce incidence of *Clostridium difficile* infection. Culturing bacteria can take days; therefore non-targeted broad-spectrum antimicrobials are often used first before sensitivities are known. Rapid diagnostic tests have been developed, which can help doctors target bacteria more quickly [5]. Group A beta-haemolytic streptococcus (GABHS) is the most common bacterial cause of pharyngitis [82]. Throat swab culture is the gold standard for diagnosis but results take one to two days. Rapid antigen detection testing gives results in 10–15 minutes (specificity greater than or equal to 95%, sensitivity greater than or equal to 90%). Madurell *et al.* showed that the use of rapid antigen detection tests to identify GABHS in patients with acute pharyngitis helped doctors identify target bacteria and devise a rational treatment approach. Procalcitonin (PCT) can be used as a biomarker for bacterial infections [83]. Burkhardt *et al.* compared PCT-guided antimicrobial treatment to standard care in patients with an acute respiratory tract infection. They showed that PCT-guided treatment reduced the antimicrobial treatment rate by 41.6% [84].

#### 2.3.3. Antibiotic Surveillance and Stewardship

The choice of antimicrobials is generally based on guidelines, which include knowledge of surveillance of bacterial susceptibility, and resistance to antimicrobials in different regions and countries. Targeted programs of active surveillance can potentially be used to guide empirical therapy [85]. Different types of surveillance used include governmental, institutional, industrial, and pharmaceutical programs, but there will inevitably be a time-lag between the results of surveillance systems and decision making in clinical contexts [86]. Antibiotic stewardship is coordinated interventions that aim to measure and improve antimicrobial usage. This includes the promotion of appropriate selection of antimicrobials, dosage, route, and duration of therapy.

#### 2.3.4. Antimicrobial Cycling

Antimicrobial cycling is scheduled rotations of antimicrobial usage between drugs with a similar spectrum of activity. The principle is that the more often an antimicrobial is prescribed, the more resistance will develop against it, and withdrawing antimicrobials for a time will remove the selective pressure, allowing resistance rates to stabilize or fall. It is important to note that the restriction of sulphonamide use nationally in the UK did correlate with a reduction in the resistance of sulphonamide-resistant *Escherichia coli* [87]. Bennett *et al.* reported an improvement in the antimicrobial susceptibility profile of Gram-negative organisms (*Pseudomonas aeruginosa*, *Escherichia coli*, and *Klebsiella pneumoniae*) in a surgical intensive care unit compared to the medical intensive unit after the implementation of rotation of piperacillin/tazobactam, imipenem/cilastin, ceftazidime, and ciprofloxacin as the primary antibiotic used to treat suspected gram-negative infections every month [88]. However, a review of both clinical trials and mathematical modelling studies examined the efficacy of antimicrobial cycling to prevent resistance and reported there was not evidence to support the implementation of antimicrobial cycling as a strategy for reducing antimicrobial resistance in hospitals [89].

## 3. Conclusions

Three core strategies to extend the lifespan of our current antimicrobials are: (i) reducing usage of antimicrobials; (ii) better use of antimicrobials; and (iii) infection control. This review examines the literature relating to the second strategy, to use antimicrobials more appropriately, concentrating on three areas: pharmacokinetics/pharmacodynamics, combination therapy, and appropriate choice of antimicrobials.

For *mycobacterium tuberculosis* the use of multi-drug combination therapy is an established strategy being used to prevent resistance to treatment. Furthermore, it has recently been suggested that the efficacy of the core anti-tuberculosis drugs, isoniazid and rifamycins, is determined by peak drug concentrations. This may mean that tuberculosis regimes can be shortened and, if so, an improvement in adherence would hopefully mean a reduction in resistance to treatment. 

For other bacterial infections, strategies to use antimicrobials in a way that will reduce resistance development are yet to be clearly established. We hope that the subject areas we have outlined in this article can be developed to help us to use our existing antimicrobials in ways that will extend their lifespan by preventing antimicrobial resistance.
